# Supplementary material for: Bringing atom probe tomography to transmission electron microscopes
Source: Nat Commun. 2024 Nov 14;15:9870. doi: 10.1038/s41467-024-54169-2 (PMC11564830; doi:10.1038/s41467-024-54169-2)
Supplement: Supplementary file 1 — Supplementary Information [file 41467_2024_54169_MOESM1_ESM.pdf]

Supplementary information of the article

Bringing Atom Probe Tomography to Transmission Electron Microscopes

Gerald Da Costa[1], Celia Castro[1], Antoine Normand[1], Charly Vaudolon[1], Aidar Zakirov[1], Juan Macchi[1], Mohammed Ilhami[1], Kaveh Edalati[2], François Vurpillot [1], Williams Lefebvre[1]

[1]Univ Rouen Normandie, INSA Rouen Normandie, CNRS, Normandie Univ, GPM UMR 6634, F-76000 Rouen, France

[2]WPI, International Institute for Carbon-Neutral Energy Research (WPI-I2CNER), Kyushu University, Fukuoka, Japan

Part 1 – Preliminary proofs of concepts

Results presented in this section were obtained with an APT-TEM holder performing at room temperature in a JEOL 2010 equipped with a LaB_6 cathode.

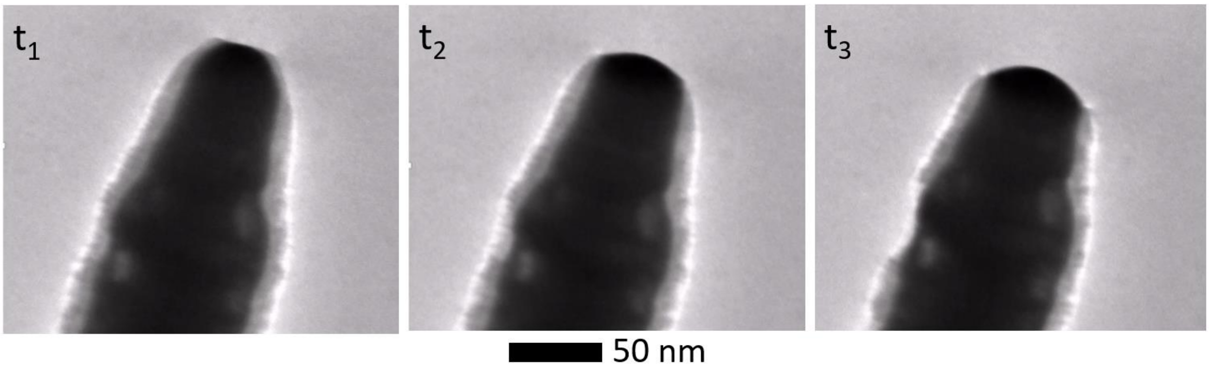

Figure S 1 : Field evaporation sequence of a W APT needle observed in bright field TEM mode. Images were recorded at various times such as $t_1 < t_2 < t_3$. The tip is covered with a layer of contamination when the cold trap is not used. A continuous potential varying from 2430V to 2600V is applied to the sample. The same tip is seen at different times t_i of its field evaporation.

An animated sequence of the evaporation sequence of evaporation of Figure S 1 is provided in the **Supplementary movie 1.avi**. The voltage applied to the specimen was increased from 2 430V to 2600 V from the first image to the last one and the specimen height was adjusted to compensate for the defocussing induced by its polarization. One can clearly see how specimen apex geometry evolves during the evaporation sequence. No liquid nitrogen trap was available when this sequence was recorded. This gave the opportunity to notice the difference of stability under high electric field

between the contamination layer and the metal. This could be simulated and the corresponding movie is available in the supplementary information. It is hence demonstrated here that access to the dynamic shape evolution of an APT specimen is possible in TEM.

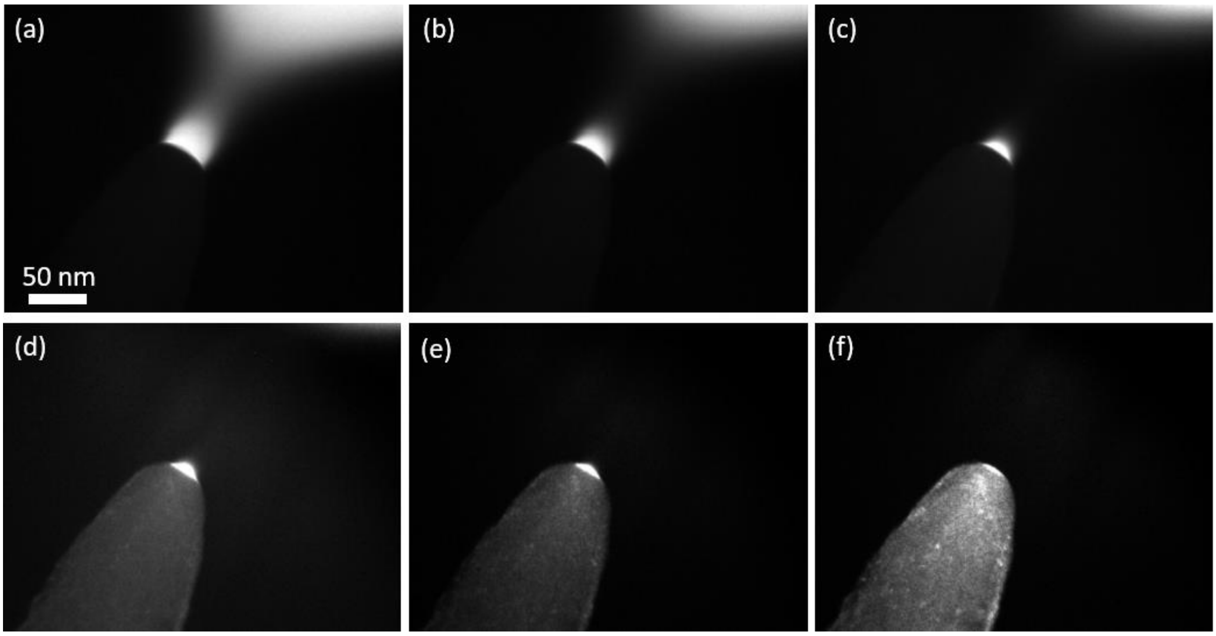

Figure S 2 : Dark field TEM images performed with the application of a 2.7 kV voltage to an aluminium based specimen.

The mapping of the electric field in the vicinity of a polarized APT specimen in JEOL 2010 could be achieved at least qualitatively using the dedicated APT-TEM holder by simply performing dark field TEM imaging with appropriate apertures, as shown in Figure S 2. The electric field distribution at the tip apex generates deflections of the electron trajectories, which result in a continuous shift of intensity in the back-focal plane of the objective lens. This imaging mode simply exploits the concept of electrostatic field-induced shift of diffraction patterns used in differential phase contrast microscopy [1,2], but in TEM mode. In Figure S 2, from images (a) to (f), the deflection of electrons used for the image is gradually increased, the highest deflection corresponding to the areas of largest electric field experienced by electron beams along their trajectory. The last dark field in (f) coincides with a reflection of the Al reciprocal lattice.

Part 2– Supplementary data obtained with APT mounted on a standard (S)TEM

The (Scanning) Transmission Electron Microscope used for this instrumental development has been delivered by JEOL company without any prior modification (Figure S 2). The microscope is a JEOL F2 equipped with a Schottky field emitter and a dry pumping system. Both TEM and STEM observations were performed on APT specimens with this instrument at 200 kV.

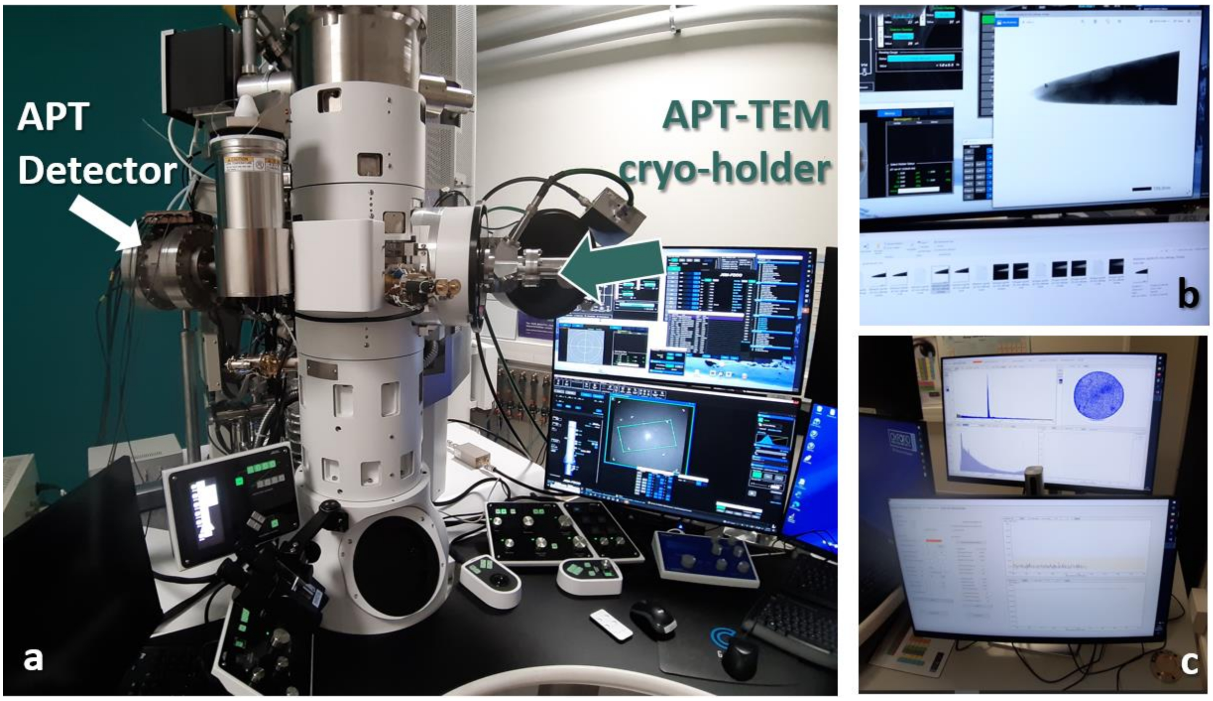

Figure S 3 : (a) JEOL F2 microscope equipped with an APT-TEM cryo-holder and APT detector both designed at GPM. (b) BF-STEM of a specimen and (c) sequence of acquisition of an Al-alloy.

The APT-TEM holder designed at GPM (Figure S 4) is compatible with the JEOL F2 and a large variety of JEOL microscopes of present and previous generations. As indicated in Figure S 3, the APT-TEM can be mounted, polarized and cooled on the microscope. In the alignment of the holder, the APT detector is mounted at the back of the column.

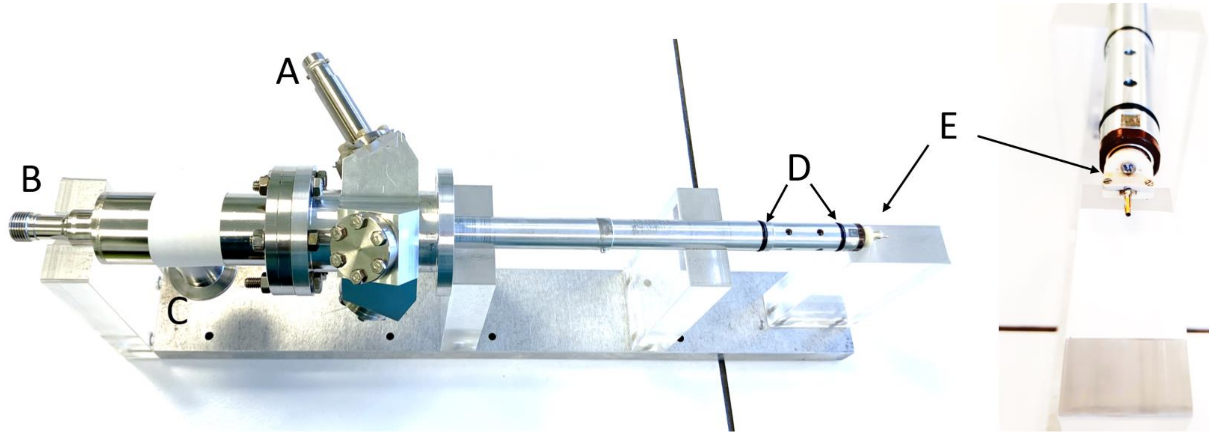

Figure S 4 : Two views of the APT-TEM holder. A: Double connection for DC and pulse voltage. B and C: Entrance and exit of liquid nitrogen, respectively. D: Double O-ring JEOL design to fulfil a complete compatibility with JEOL TEM F200 goniometer. E: specific ceramic piece, made of alumina (and a plate of peek) to ensure the specimen assembly, the adapted connection to the nitrogen circulation and the insulation from the link used to apply high voltages.

In Figure S 4, the different parts of the APT-TEM holder are visible in the assembled holder. The left part is the cryo-cooling part, which goes to the extremity of the holder. It is connected to the main body of the holder, where the high voltage connexion (A) is present. It is also connected to the ceramic part, on which the APT specimen is inserted.

The Atom Probe Tomography detection system mounted on the JEOL F2 is an aDLD (advanced delay-line detector) detection system[3]. It is made of an assembly of 2 microchannel plates (MCP) coupled to a 2-dimensional independent delay line anode. When atoms hit the detection system, the electron clouds created from the MCP assembly generate electrical signals that propagate along the 2 independent delay lines. Fast analogue-to-digital converter (ADC) digitizers are used to collect X and Y electrical signals on the lines. An advanced delay line detector algorithm is applied to extract timing information from signals in order to define the number of detected atoms and then calculate the related position and mass of charge values.

An evaporation sequence has been recorded in bright field TEM mode on the same Fe-51.4Cr(at%) alloy and same thermomechanical state as the one detailed in Figure 3 of the article. The full evaporation sequence recorded for a variation of DC voltage from 4.0 KV to 5.01 kV is shown in the movie **Supplementary movie 2.avi**. A length of about 270 nm was field evaporated during this sequence. Figure S 5 displays some of the images recorded during the field evaporation, which showcase the gradual variation of curvature of the specimen apex. Accounting for such variations for APT reconstructions is an opportunity offered by the new instrument combining APT and (S)TEM.

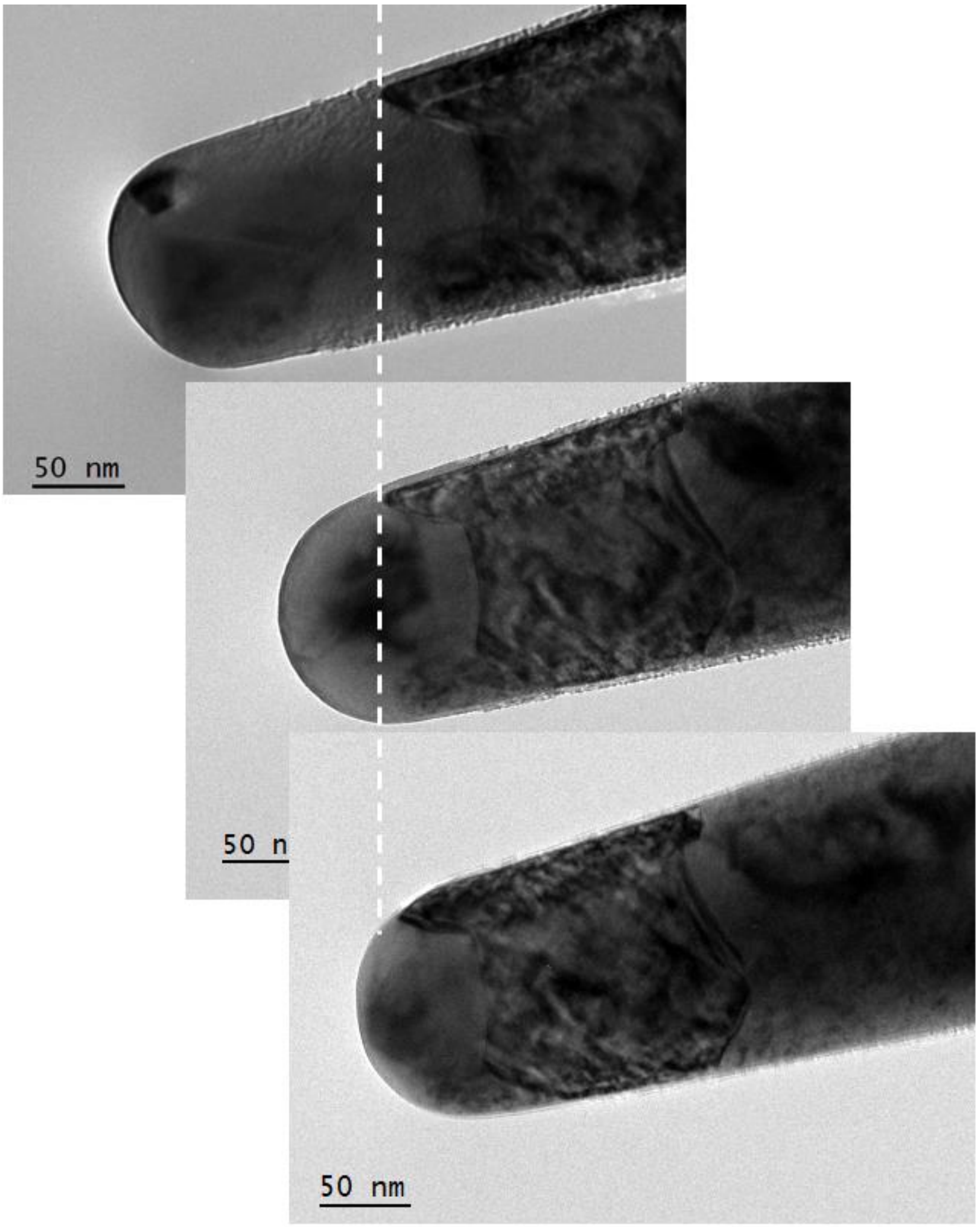

Figure S 5 : Images extracted from a field evaporation sequence of the Fe-51,4at% ultrafine grain alloy recorded at room temperature in BF TEM mode. These images were recorded at the constant voltage of 4.86 kV (first image on top). The white vertical line indicates a fixed spatial reference for the tip.

Figure S 6 and Figure S 7 provide additional information about the indexation of crystal orientation performed in Figure 3 on the article. In Figure S 6, for each column of the diffraction pattern is shown on top, followed by its automated indexation. In Figure S 7, respective stereographic projections for grains 1 to 3 are shown. The corresponding projected orientation of the body centred cubic crystal in respective grains 1 to 3 is displayed.

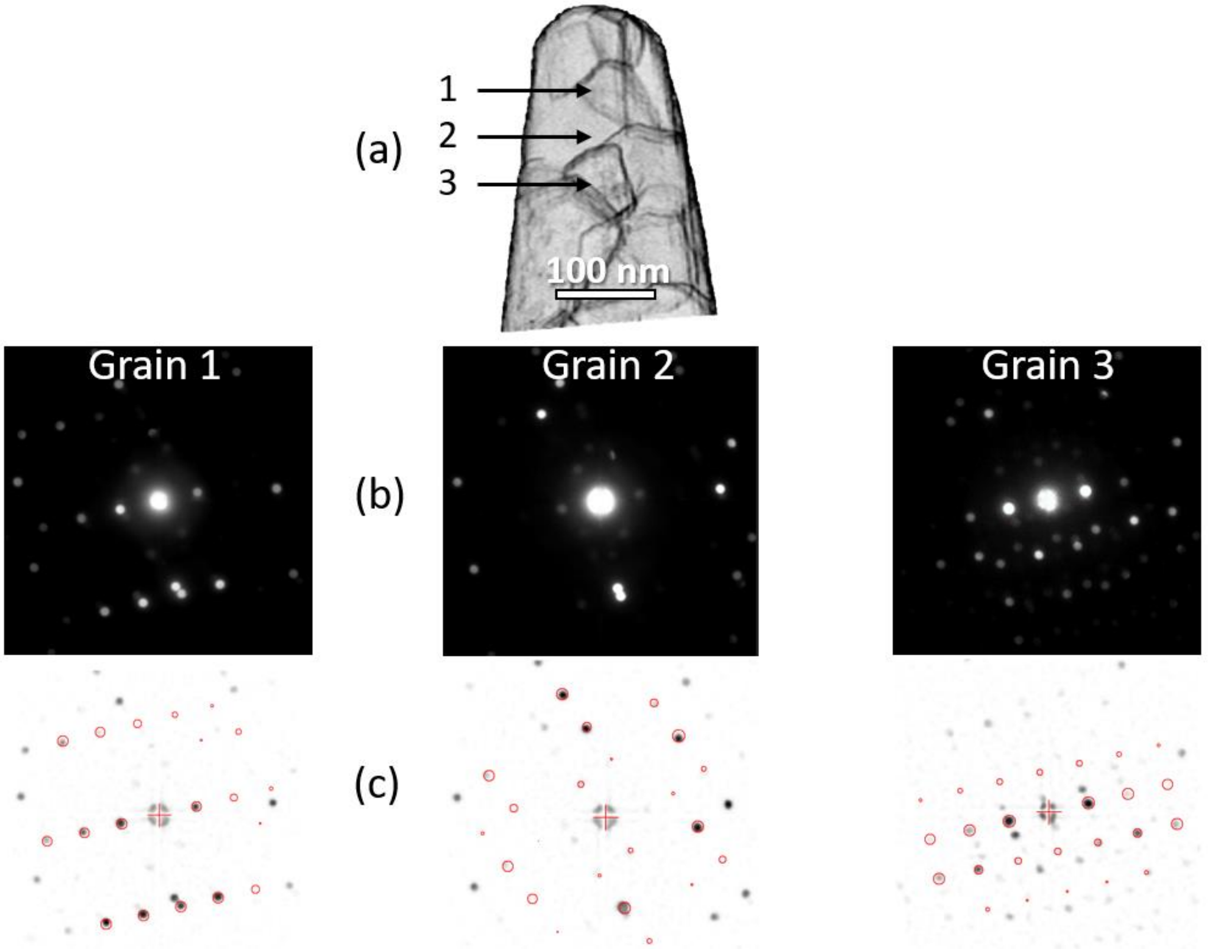

Figure S 6 : Indexation of diffraction patterns obtained in grains 1, 2 and 3 performed with the automated crystal orientation and phase mapping methodology[4]. (a) Location f the grain in the APT specimen. (b) Diffraction patterns recorded in each grain location. (c) Overlap of the recorded diffraction patterns with the diffraction pattern automatically detected with the automated crystal orientation and phase mapping methodology[4] (red circles).

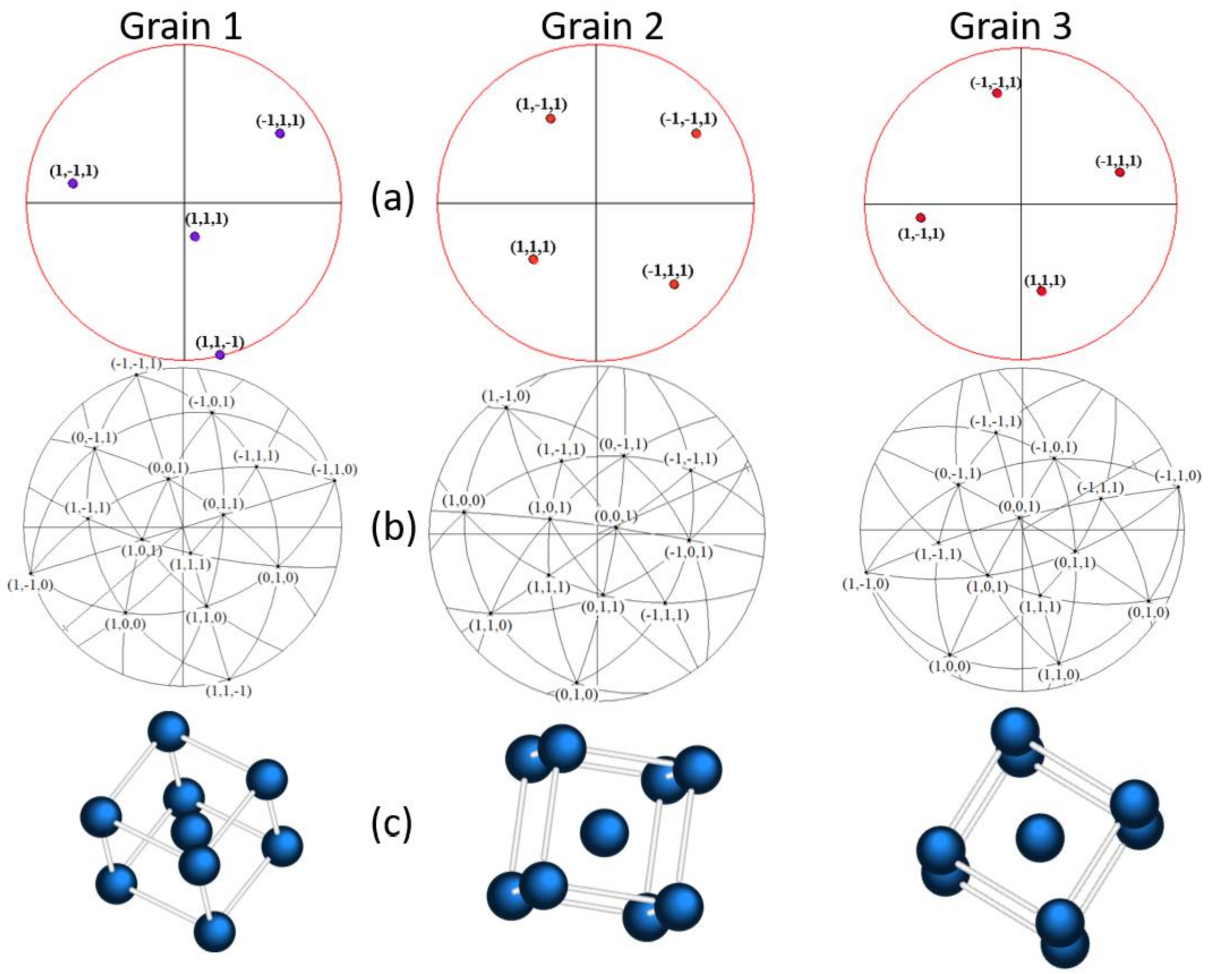

Figure S 7 : Details about the automated crystal orientation presented in Figure S 6. (a) Automated crystal orientation represented in a stereographic projection. (b) Construction of a stereographic projection for the same orientation. (b) Projection of the crystal structure for each grain orientation.

Supplementary References.

1. Shibata, N. *et al.* Differential phase-contrast microscopy at atomic resolution. *Nat. Phys.* **8**, 611–615 (2012).

2. Müller, K. *et al.* Atomic electric fields revealed by a quantum mechanical approach to electron picodiffraction. *Nat. Commun.* **5**, 1–8 (2014).

3. Da Costa, G., Vurpillot, F., Bostel, A., Bouet, M. & Deconihout, B. Design of a delay-line position-sensitive detector with improved performance. *Rev. Sci. Instrum.* **76**, (2005).

4. Rauch, E. F. & Veron, M. Automated crystal orientation and phase mapping in TEM. *Mater. Charact.* **98**, 1–9 (2014).
