# Supplementary material for: Bringing atom probe tomography to transmission electron microscopes
Source: Nat Commun. 2024 Nov 14;15:9870. doi: 10.1038/s41467-024-54169-2 (PMC11564830; doi:10.1038/s41467-024-54169-2)
Supplement: Supplementary file 2 — Description of Additional Supplementary Files [file 41467_2024_54169_MOESM2_ESM.pdf]

Description of Additional Supplementary Files

Supplementary Movie 1: Field evaporation sequence of a W APT needle observed in bright field TEM mode. The tip is covered with a layer of contamination when the cold trap is not used. A continuous potential varying from 2430V to 2600V is applied to the sample.

Supplementary Movie 2: Field evaporation sequence of the Fe-51,4at% ultrafine grain alloy recorded at room temperature in BF TEM mode. The full evaporation sequence was recorded for a variation of DC voltage from 4.0 KV to 5.01 kV. A length of about 270 nm was field evaporated during this sequence.
